# Examining Cross-Age Experiences in a Distance-Based Intergenerational Music Project: Comfort and Expectations in Collaborating With Opposite Generation Through “Virtual” Exchanges

**DOI:** 10.3389/fmed.2018.00214

**Published:** 2018-08-13

**Authors:** Melita J. Belgrave, Daniel J. Keown

**Affiliations:** ^1^School of Music, Herberger Institute for Design and the Arts, Arizona State University, Tempe, AZ, United States; ^2^Dana School of Music, Youngstown State University, Youngstown, OH, United States

**Keywords:** intergenerational, older adults, music performance, music therapy, attitude, distance collaboration, virtual exchange

## Abstract

There has been an increase in the number of music-based intergenerational programs conducted by music therapists as part of wellness and intergenerational music therapy programs. Research has shown that intergenerational music therapy programs have improved cross-age attitudes, interactions, and older adults' psychosocial well-being. Anecdotal evidence indicates that one of the challenges for creating music-based intergenerational programs is finding agencies that serve younger and older generations within close proximity to one another. We sought to remedy this problem with the integration of “virtual” technology. The purpose of this study was to examine changes in cross-age comfort, expectations after experiencing “virtual” exchanges, and preconceived notions of older and younger persons enrolled in a distance-based intergenerational project. A secondary purpose was to determine what intergenerational project factors were most enjoyable for older and younger participants. Eighteen older adults (61 through 79 years old) from an intact music-therapy choir along and 14 younger children from an intact community choir (9 through 14 years old) served as participants for the current study. All participants experienced the 4-week distance-based intergenerational program that consisted of: (a) two group “virtual” exchanges, (b) two reflective journals related to the “virtual” exchanges, (c) an in-person half-day music-therapy intergenerational workshop, and (d) a joint performance. Cross-age comfort, preconceived notions, expectations, and preference factors were examined through pre-test and post-test measurements. Results indicated an increase in older adults' comfort level collaborating with children after participation in the intergenerational music project. The majority of younger (64%) and older (69%) participants indicated that their preconceived notions about the other generation were different and positive from what they initially thought prior to the project. Both generations indicated an improvement in cross-age attitudes and interactions due to the collaboration process (“virtual” exchange, workshop, and joint-performance) and the cross-age interactions during the project. These findings suggest that music therapists can foster cross-age interactions and relationships between generations that are not within close proximity of one another by using a combination of “virtual” and “live” interactions as an intervention for enhancing the overall quality of life among older adults.

## Introduction

There has been a rapid increase in the aging population worldwide, and this trend will continue for the next 30 years. Currently older adults, aged 65 and over, comprise 8.5% of the population in the world. It is estimated by the year 2050, this number will double and older adults will comprise 16.7% of the population, resulting in 1.6 billion older adults worldwide (1). With the rise in the number of older adults living either in the community or in facilities for people who are aging, it is important for healthcare professionals to offer a variety of services and creative programs that enhance older adults' well-being.

One way that older adults can maintain their well-being is to participate in music-based intergenerational programs. Research has shown that participating in music-based intergenerational programs can improve cross-age attitudes, cross-age interactions, and older adults' psychosocial well-being (2–8). Music-based intergenerational programs have been conducted with a wide range of younger and older generations. Younger generations have included children in preschool, elementary-aged children, younger persons in middle-school, and high school, as well as young adults and adults enrolled in higher education. Older generations have included active older adults living in the community and older adults living in facilities who are diagnosed with dementia, Alzheimer's disease, and other age-related illnesses (5, 9–15).

There are four models commonly used in intergenerational programs: (1) younger and older generations engaged in combined learning programs, (2) younger generations serving older generations, and (3) older generations serving younger generations, and (4) younger and older generations engaged in recreational activities. The selection of the model is often chosen by the music practitioner and based on the goal of the program, the chronological age of each generation, and the needs and abilities of each generation. Some music-based intergenerational programs have occurred as group therapeutic sessions where younger and older generations engage in music learning together. In these settings both generations participate in singing of familiar songs and newly learned songs, structured conversation, instrument playing, and moving to music interventions (2, 16). Other music-based programs have employed older generations serving younger generations, where an older person provides piano accompaniment for a school choir. Additionally, music-based programs have used younger and older generations engaged in recreational activities where individuals join small ensembles together or perform music in intergenerational dyads. Results from research conducted in these various settings show that older adults perceived improved attitudes toward younger persons, improved quality of life, and increased social interaction were benefits from participating in intergenerational programs (6).

Many programs have been conducted as intergenerational performance ensembles that bring together younger generations and older adults in a learning together model where both generations learn new and familiar music together. The program usually ends with a culminating intergenerational performance, featuring the selections that both groups learned together (4, 5, 8, 17). Researchers have found that both generations enjoy performing with each other, and perceive the performance to be better because of the opposite generation or the intergenerational component. Additionally, participants reported improved cross-age attitudes, and understanding of others. Finally, researchers have found improvements in younger persons' willingness and comfort in working with older generations in a music setting (4, 5, 8).

One of the challenges for creating music-based intergenerational programs is finding agencies that serve younger and older generations within close proximity to one another. Researchers have discovered that prior experiences with collaborators can reduce barriers as a result of distance between two parties (18). Although face-to-face communication seems to be the preferred form of communication with family members among older adults (19), communication through technology has recently expanded communication for a population that is typically socially separated from the general public (20). Audiovisual technology and video chat (e.g., *Facetime, Skype*) has also been a recent tool used to share experiences with family members outside a regional location whenever they occur (21). This integration has provided new opportunities for grandparents to develop and maintain relationships with their remote grandchildren (22). Perhaps the integration of audiovisual technology could be a viable and relevant route toward developing relationships between the older and younger generations during the collaboration.

Another challenge is finding times in both facility calendars to schedule the intergenerational program. The majority of the music-based intergenerational programs found in the research literature have been conducted with groups that are within close proximity and occur for several sessions in person. Music therapists have expressed interest in intergenerational programming, but have informally cited lack of two cooperating facilities (younger generation and older generation) within close proximity to one another as a deterrent for creating an intergenerational program. We proposed a remedy to this problem by using simple and inexpensive technology to facilitate these types of programs.

The purpose of this study was to investigate how a combined “virtual” and “live” 4-week intergenerational music collaboration impacted children and older adults' cross-age comfort, expectations after experiencing “virtual” exchanges, preconceived notions, and enjoyable factors in collaborating with the opposite generation. Research questions included:

1. Will participants' cross-age comfort change after participating in a music-based intergenerational project?
2. What expectations about the other generation or the collaboration project emerged from participants after experiencing the “virtual” exchange?
3. Will participants' preconceived notions about the other generation be similar or different from what they initially thought prior to the project?
4. What were the most enjoyable factors of the project among younger and older generations?

## Methods

### Participants

Eighteen older adults (13 female, 5 male) from an intact older adult music-therapy choir and 14 children (9 female, 5 male) from an intact community children's choir served as participants for the study. Ages for older adult participants ranged from 61 through 79 years of age (*M* = 69.29, *SD* = 5.49), while ages for children participants ranged from 9 through 14 years of age (*M* = 11.29, *SD* = 1.20). A distance of approximately 500 miles separated the two choir's home location in Midwestern regions of the United States.

The older adult music-therapy choir specialized in learning and performing pop/rock music with the assistance of the director of the group and music therapy university students as part of a clinical course. This group would normally rehearse once a week at a local community center. The children's choir traveled to the older adult choir's residing city for a 3-day tour. Founded in response to the African-American civil rights movement, the children's choir annually seeks out tours and collaborations that allow students exposure to diversity through singing experiences. The children's choir inquired about collaborating with an older adult population and was introduced to the current older adult choir at their regional location. The choir directors, children's choir staff, and investigators worked together to organize the initial framework of the exchange a few months prior to the start of the 4-week collaboration.

### Procedures

A 4-week intergenerational program was developed by the investigators and implemented with the assistance of undergraduate music therapy, music education, and graduate music therapy majors as part of a course's curriculum. All participants experienced a 4-week intergenerational younger and older generation learning together model that consisted of: (a) two group “virtual” exchanges, (b) two reflective journals related to the “virtual” exchanges, (c) a “live” in-person half-day music-therapy intergenerational workshop, and (d) a joint performance. The “virtual” exchange and reflective journals related to the “virtual” exchanges occurred within 1 month before the two choirs collaborated in person at one site for the “live” portion of the collaboration.

#### “Virtual” collaboration

One goal of the intergenerational collaboration project was to use audiovisual technology to assist in preparing for the “live” collaboration and performance due to a distance factor that precluded the two choirs to collaborate in person prior to the main workshop and performance. Ultimately, the final “live” performance would include each choir performing their own set of selections for each other followed by the performance of two combined selections to end the concert.

Since the choir's normal rehearsal dates and times did not allow for real time virtual visual streaming, audio-visual recordings transferred via *Dropbox* were used to virtually collaborate between the two choirs. The “virtual” collaboration began with participants completing a pre-test on cross-age comfort, preconceived notions about the other generation, and expectations regarding the collaboration at their respective home locations. All “virtual” exchanges occurred during the choir's normally scheduled rehearsal. Following the pre-test, each choir video-recorded an introduction on the history of their choir, where they rehearsed, and shared a performance of a piece that they felt represented their choir. The adult choir's representative piece was “Roar” by Katy Perry (2013) whereas the children's representative piece was “Dancing in the Street” by Martha and the Vandellas (1964)^1^. Each respective choir director selected the representative piece for this project. This initial performance of the representative piece and description of the selection was in effort to initiate a musical collaboration virtually between the two groups. At a later date, each choir watched the other choir's video recorded message during rehearsal. Following the viewing of Video #1, choir participants were asked to complete a journal entry in response to the video and anticipated collaboration. The purpose for journal responses were twofold: (1) to structure constructive reflection based on the “virtual” exchange, and (2) longitudinal data collection.

During the following week, a second video recording was created integrating what individual participants were anticipating about their live interaction, individual personal messages about background and why they like music and singing, and a teaching demonstration from each choir. For the teaching demonstration, the children's group taught the older adult group “Count on Me” (2010) by Bruno Mars. The older adult group taught the children's group “You've Got a Friend in Me” from *Toy Story* by Randy Newman (1995). Both pieces were selected collaboratively between both choir directors. Each group could then rehearse with the audiovisual recording in order to get used to singing with each other. In addition, a few individuals from each group shared their thoughts of the other choir's performance from Video #1 and provided their thoughts and anticipation regarding their visit and collaboration.

Each choir viewed the second video recording and asked to complete Journal #2 by answering the same three questions asked in the first journal entry. All pre- and post tests, and journal entries were collected and coded to track individual responses across time.

#### “Live” collaboration

The live collaboration involved a half-day workshop collaboration and rehearsal between the adult choir and children's choir followed by a performance for the community as part of the children's choir tour at the older adult choir's regional location. The workshop included four sessions organized and facilitated by undergraduate and graduate music therapy and music education students under the guidance of the two investigators.

As part of a collaboration between a music therapy and music education course, university students had the task of structuring and implementing the “live” exchange segment of the intergenerational music collaboration. Weeks before the “live” exchange the investigators, who were also the university instructors for these courses, facilitated a brainstorming session that allowed students to form groups and select interactive activities in alignment with two of the shared selections and two new pieces that could be taught by rote. Although the university students were provided liberties in structuring the activities, each group was required to integrate structured conversation and music making within each session in effort to foster participatory music-making between both generations (15). Students used newly acquired skills from the music therapy and music education course to inform their decisions on such factors as vocal range/ability, musicianship, conducting, performing, instruction, and assessment.

On the day of the “live” exchange, several groups were randomly formed comprising of a mixture of adults and children, which varied for each session. University students facilitated all planned sessions during this exchange. Session #1 used the teaching and performance of the song “Everybody Loves a Saturday Night” to guide singers in introducing themselves and facilitating structured conversation related to what each singer enjoyed doing on Saturday nights. Using a fill in the blank song writing worksheet, the participants wrote their answers, and the university music students facilitated the singing of new lyrics to include the types of activities participates enjoyed doing on a Saturday night.

Session #2 used the teaching and performance of “Yakety Yak” by the Coasters (1958) as a guide for introducing themselves and facilitating structured conversation related to the types of chores they currently do or did when they were younger. In a humorous fashion, groups would sing the song for the other members, with the assistance of background tracks, and acted out the physical gestures of completing the chore that each singer identified.

Session #3 began by rehearsing one of the combined pieces, “You've Got a Friend in Me.” After a short rehearsal, structured conversation ensued as singers shared the types of things that they like to do with her/his friends. After a short conversation, the small groups were collapsed into a larger group and individual singers introduced other singers from their small groups by identifying what they like to do with their friends. The session concluded by incorporating movement in the performance of the piece, informed by some of the actions discussed in conversation.

Session #4 began by rehearsing the other combined piece, “Count on Me.” Facilitators of the session continued by teaching singers various rhythms that align with the piece and proceeded to have the singers perform these rhythmic patterns on non-pitched instruments with the song. Structured conversation developed during a break in performance where singers shared specific characteristics and qualities that good friends should possess. Using a “call-stop-listen” system, periodic announcements were made to reconfigure new pods of individuals and repeat this activity with a new group of individuals. The session concluded with a performance of the piece accompanied by percussion instruments.

Following a combined lunch, the choirs performed for each other and a community audience, including the two representative pieces and two combined selections to conclude the concert. After the concert, singers from both choirs had some free time to visit with each other before departing for the day. Prior to departure, singers from both choirs completed the post-test survey.

### Data collection and analysis

Cross-age comfort, preconceived notions, and expectations were measured through a researcher-developed tool that was given as a pre-test and post-test measure as part of this feasibility study. Cross-age comfort was measured on a 6-point Likert-type scale (1 = not comfortable; 6 = very comfortable) indicating level of comfort in collaborating musically with the other generation and whether participants had experiences collaborating musically with the other generation of individuals. Preconceived notions, expectations, and enjoyable factors were measured through open-ended questions; and a manifest content analysis was conducted on open-ended responses. Furthermore, participants were asked to answer three writing prompts after viewing each “virtual” video-recorded exchange in the form of journal responses.

This study incorporated a summative content analysis to identify frequency of emerging themes found in participants' open-ended written responses across a 4-week time frame (23). The open-ended responses offered insight into personal preconceived notions about the other generation, expectations after experiencing the “virtual” exchange, “live” collaboration expectations, and the enjoyable factors related to the other generation singers and the intergenerational collaboration process. At the end of the project, two researchers independently analyzed written responses by identifying emerging themes and incorporating a manifest content analysis by counting the frequency of emerging themes for further quantitative analysis (24). In determining the total frequency of themes per question, it was possible for more than one theme to be identified per question during analysis. Therefore, it was deemed inappropriate to calculate statistical analyses and instead offer descriptive analyses of the results. After the initial independent analysis, the two investigators confirmed emerging themes and began a quantitative analysis for each question.

This study was submitted and approved by the university's Institutional Review Board (#14-063) prior to any recruitment of participants or data collection.

## Results

### Changes in cross-age comfort

Each group of participants (older adults and children) completed a pre-test and post-test based on their attitudes and perceptions of the opposite generation. Using a 6-point Likert-type scale (1 = not comfortable; 6 = very comfortable), participants rated their level of comfort in collaborating with the opposite generation before and after the collaboration project. Data within subjects were treated independently due to absenteeism for both tests. Therefore, a group's pre and post-test responses were calculated instead of individual pre and post-test responses.

A Mann-Whitney test indicated a significant difference in older adults' level of comfort in collaborating with children before and after the intergenerational music collaboration project, *Z* = 2.87, *p* < 0.05 (*r* = 0.46). The moderate effect size could also suggest a possible practical significance. Adults reported a higher comfort rating on the post-test compared to the pre-test. A second Mann-Whitney test indicated no significant difference in children's comfort level in collaborating with older adults after the project, *p* > 0.05. However, similar to the older adults, children participants had a noticeably high comfort level rating prior to the start of the project (see Table 1).

**Table 1:** Pre-test and post-test comfort level ratings.

	**Pre-test**		**Post-test**	
	**Median**	**Range**	**Median**	**Range**
Children	5	2	6	2
Older Adults	5.5	4	6	1

### Expectations after the “virtual” exchange

Singers from both choirs completed a journal following each “virtual” video recording exchange. Video #1 included background information on the choir and the explanation and performance of one song that the choir felt best represented their group. Video #2 included individual reactions toward the choir's first video recording, what singers anticipated upon the visit, continued background information on individual singers, and a teaching demonstration of a song. In response to watching the adult choir perform “Roar,” one child stated:

When I saw the “Roar” video, I thought it was very nice that you guys were doing a popular song, and I thought you guys sounded really good, and I thought that it was just nice that you guys would do that, and I thought, and I was really excited about it.

In response to the children's choir, one older adult indicated her impression on the sound of the choir:

I just saw the children's video …. and I have to say, well our words would be cool, but in their words “very sweet” [makes quotation mark gestures when saying “very sweet”]. I thought that the entertainment—the songs they sang and the way they moved and danced for singing was spectacular—very upbeat. Intelligent and wonderful children. [makes thumb up gesture to the camera]. We look forward to meeting you.

Another older adult wanted to prepare the children on what to expect from the older adults in terms of their maturity:

This is my first year with this choir and I'm really having a lot of fun with it. Um, your choir sounds wonderful. I can't wait to meet you guys and I don't think you have to worry about us being too mature because we are pretty immature. So, see you in a bit—bye-bye [makes big circular hand gesture as a wave gesture].

Both journal responses asked the same three questions: (1) What did you think of the older adults [or children] in the video? (2) What are you looking forward to when collaborating in person with the adult choir [or children's choir]? (3) What are you unsure about in collaborating with the adult choir [or children's choir]? From the written responses, participants focused on music, non-music, or collaboration topics when answering these questions. Therefore, an analysis for each question quantified the frequency of these topics.

A descriptive analysis indicated a noticeable shift from writing about music responses to non-music responses over the course of the journal cycle among older adults, whereas children remained steady in focusing on music responses for both journal entries when answering the question on what they thought about the other generation in the video (see Table 2). The older adults provided musically driven comments in Journal #1 such as:

The children have beautiful voices. I was surprised by the volume considering the size of the group.

**Table 2:** Frequency distribution of referenced topics from journal response questions after “virtual” exchanges.

**Question 1: what did you think of the older adults/children in the video?**	**Music (%)**	**Non-Music (%)**	**Collaboration (%)**	**No Response (%)**
Adults–Entry 1	45	41	14	0
Adults–Entry 2	28	56	12	4
Children–Entry 1	52	22	26	0
Children–Entry 2	44	41	15	0
**Question 2: what do you look forward to in collaborating with the older adults/children?**	**Music (%)**	**Non-music (%)**	**No response (%)**
Adults–Entry 1	30	70	0
Adults–Entry 2	26	74	0
Children–Entry 1	74	27	13
Children–Entry 2	47	53	0
**Question 3: what are you unsure about in collaborating with older adults/children?**	**Music (%)**	**Non-Music (%)**	**No Response (%)**
Adults–Entry 1	28	28	44
Adults–Entry 2	26	21	53
Children–Entry 1	25	19	56
Children–Entry 2	27	33	40

However, the second journal entry focused more on the dispositions of the children:

They are all very cute

The kids are very articulate. I was impressed.

I felt like the children were speaking directly to us. They expressed themselves so well. Their choir has a great energy and are well rehearsed. They pay attention. So nice!

When asked about what they were looking forward to when it came time for the “live” collaboration with the other generation, older adults seemed to focus on non-musical aspects throughout both journals, whereas children began more focus on music characteristics for the first journal entry, but shifted closer to non-music aspects after viewing the second video recording. When the adults did reference musical aspects, they were interested on how the children could help them with their music technique and performance:

A good lesson in showing us their poise when performing ahead of older adults.

Catch some of their enthusiasm and love of music.

It's always a joy to perform with young people. It will be so much fun and I look forward to learning from them.

Another adult participant seemed to be eager in teaching and nurturing the children upon their arrival while hoping to find commonalities between the children and their own grandkids:

I'm looking forward to helping the kids be relaxed, happy to be here and help them have an enjoyable experience.

I'm looking forward to meeting the children. They are the age of some of my grandkids and we can have fun together.

The desire of making new friendships found its way in some of the responses as well. Overall, the “virtual” exchanges seemed to offer anticipatory understanding for the older adults in establishing a sense of the children's dispositions, music skill, and performance effort while instilling a sense of learning and developing friendships when it came time to collaborate with the children.

Although both choirs seemed to state that there wasn't much that they were unsure about when the “live” exchange would occur, a few participants indicated both music and non-music aspects that they were unsure about. The children seemed to be unsure on how they would function within the collaboration:

I am unsure about my behavior.

I am unsure about how we will collaborate with them. How will we get to know them? How long will we be with them for?

The few older adults who answered this question seemed to wonder how they would compare to the children:

They seem very well trained and have great voice. Will we be able to keep up with them?

Makes me self-conscious about the movement limits.

However, even with a few reservations among the older adults, they seemed to be determined to approach the project with effort:

They are so good. I fear we will not measure up (sad face). But we're in it!

### Preconceived notions about the other generation

Participants wrote about their preconceived notions on the other generation and expectations for how various facets of the collaboration would turn out through open-ended questions from pre/post-test surveys. Pre-test analyses found that the majority of written statements regarding preconceived notions on the other generation were positive. However, it seemed as though the children's responses were directed more on their preconceived notions about themselves, whereas the older adults preconceived notions centered more on the collaboration process with the children. One adult remarked:

I imagine lots of smiles and laughter from both groups and ultimately of [heart shape for love.

1. No germs

2. Fun

3. Free interactions to visit.

4. Play in a frolicking way.

Post-test analysis identified responses as either similar or different based on preconceived notions and whether differences were positive or negative. The majority of older (69%) and younger (64%) participants indicated that their preconceived notions about the other generation were different from what they initially thought prior to the project. From these differences, the majority of younger (79%) and older (72%) participants reported the differences to be positive at the conclusion of the project. Seventeen percent of older and 11% of children participants who originally indicated a difference in preconceived notions indicated no response as to whether the difference was positive or negative. From the preconceived difference statements made by the children, 57% of the comments focused on notions about the adults compared to different notions on the collaboration experience (29%) or differences in oneself (14%). Similarly, older adults also addressed preconceived notions about the children (56%) more often than the collaboration experience (19%) or oneself (13%). Some preconceived notions that were reported as different among the older adults were the children's dispositions while comparing their understanding of a child's developmental stages to the collaborating children:

I thought the children would be more shy. They were very good in conversation.

[I] did a lot of health assessment of well-children in schools and communities. Similar developmental stages. But unique skills, talents for the group.

Mostly, it was different than working with teenagers, but I love every minute of it! Kids have a certain magic that makes my day- Better than teenagers in many ways.

However, it seems to be important to note that some adults found the entire experience contributed to her/his overall well-being:

I didn't know how comfortable I would feel with them. They helped me to be relaxed and have a good time.

One adult also mentioned how the children were similar to what they thought in some ways due to the experiences in learning about the children through the virtual exchanges:

We had seen videos of the choir, so I knew they were a quality ensemble. But it was even more inspirational working with them than I anticipated.

For this older adult, it seems as if the “virtual” exchange provided some knowledge in preparing the older adults on what to expect of the children, but that the “live” interactions seemed to be more intrinsically inspiring.

### Enjoyable factors

Investigators independently and collectively analyzed written responses pertaining to enjoyable factors from the post-test. Three central types of response themes were discovered: (1) enjoyment of and with self, (2) cross-age interactions, (3) collaboration process (“virtual” and “live” collaborations, and performance). When a participant wrote about the process of collaborating virtually or in person, a collaboration process response was indicated. However, if the participant stated a specific interaction with the other generation, then a cross-aged response was indicated. The top two enjoyable factors for both generations were the collaboration process (“virtual” exchange, workshop, and joint-performance) and the cross-age interactions. Older adults seemed to indicate more enjoyable instances in the collaboration experience (50%) compared to the cross-aged interactions (40%), whereas children reported slightly more enjoyable instances in cross-aged interactions (44%) followed by the collaboration experience (40%). From these statements, older adults referenced non-musical experiences (67%) as enjoyable factors more often than musical experiences (33%). In one instance, an adult participant made a comparison to the children and her/his own grandchildren as an enjoyable factor. Children participants also referenced non-musical experiences (48%) more often, but only by a smaller margin compared to music experiences (40%).

## Discussion

The purpose of this study was to explore how a combined “virtual” and “live” 4-week intergenerational music collaboration impacted children and older adults' cross-age comfort, expectations after experiencing “virtual” exchanges, preconceived notions, and enjoyable factors in collaborating with the opposite generation. Results showed that older adults' comfort collaborating with children increased after participating in the intergenerational project. These findings are similar to Bowers (4), where cross-age improvements were greater for older adults than for younger persons. It should be noted that the children in this project had a high level of comfort interacting with older adults musically prior to the start of the project. The children's choir often tours across the country and outside of the country. During their tours, it is common for the choir to collaborate musically with other ensembles. Perhaps the choir members were used to collaboration whereas, the older adults had not collaborated with another music group.

Additionally, the researchers explored the most enjoyable factors of the project among younger and older generations. The top two enjoyable factors for both generations were the collaboration process (“virtual” exchange, workshop, and joint-performance) and the cross-age interactions. These findings are similar to Conway and Hodgman (5), who brought together two intact choirs for an intergenerational collaborative project. In the earlier study, both older and younger participants experienced positive outcomes of the joint-performance and perceived that the combined choir sounded better than if each choir would have performed by themselves. Participants of the earlier study also thought a benefit of the project was the cross-age interactions and understanding of the other generation.

Although the schedules of the two choirs did not allow for “virtual” interactions that were synchronous, both generations reacted positively to the interactions and cited the interactions as one of the top enjoyable factors of the project. It was interesting to notice how the older generation's writing shifted from music related topics to non-music topics over the course of the “virtual” cycle. This could suggest that the use of technology to facilitate these “virtual” exchanges could contribute to the time needed for more interaction with children in order for adults to focus on or reference non-music factors such as the children's dispositions or what the children could offer the adults during the exchange. Even more, the use of simple and free computer technology (e.g., *Dropbox, tablets, smartphones*) could be a promising vehicle for communicating, learning, and enhancing relationships between opposite generations, beyond the scope of just music exchanges.

### Future studies and limitations

Future research studies should explore the use of synchronous interactions between the generations during a distance-based intergenerational project. The current project lasted 4 weeks, which is a short duration for an intergenerational project. Future researchers should explore the benefits of an intergenerational program on younger and older persons during a program of longer duration.

Secondly, further examination is needed to collect data from a larger sample in order to achieve statistical power. One of the main limitations in the current study was analyzing responses from a single collaborative project with a small sample size. Due to the abundant amount of community children's choirs residing in each state of the United States, older adult choirs and intergenerational music ensembles may want to initiate more collaborations. Through these collaborations, improvements and alternative approaches to how the technology is integrated during “virtual” exchanges could provide a basis in establishing a “virtual” and “live” intergenerational music collaboration. Researchers could identify multiple collaborations in an effort to collect data from a larger sample of participants. These collaborations could even extend outside of the United States and across different countries.

In addition to the examination of collaboration and cross-age interaction, which participants chose to reference in open-ended responses, it could be helpful to examine with more depth how these two components contributed to their sense of self in a future study. Perhaps a study examining specific attributes of the virtual exchange that participants found meaningful could be investigated further. Furthermore, the amount and timing of intergenerational music models could warrant further examination. Since the various virtual interactions that occurred over the course of 4 weeks seemed to contribute toward the participants' wellbeing, future models could consider the benefits of facilitating these types of collaborations beyond a few interactions within a small time frame.

Finally, emerging themes found in the current study could be used to develop a more robust measurement tool. The open-ended responses allowed the researchers to discover and quantify various themes. However, it was difficult to rank order these themes based on most importance through this measurement tool. Therefore, a new measurement tool, which could be standardized, may provide more validity and reliability to data centered on comfort, attitudes, and expectations between opposite generations similar exchanges.

Overall these findings suggest that music therapists can foster cross-age interactions and relationships between generations that are not within close proximity of one another by using a combination of “virtual” and “live” interactions. These findings also suggest that music therapists can use technology to deliver structured music therapy interventions to build rapport between generations prior to an in-person meeting.

## Ethics statement

This study was carried out in accordance with the recommendations of the University of Missouri-Kansas City International Review Board (IRB). The protocol was approved by the UMKC-IRB committee in the spring of 2014. Older adult participants received a consent form indicating the purpose, risks, and human subject rights for participation. The only data collected was from participants who agreed to participate by signing the consent form. Children participants were under the age of 18 and considered minors. Therefore, children participants who wanted to participate in the study needed to gain parent/guardian signature on the consent and sign their own assent form. Again, no data was collected from children participants without receiving both parent consent and children assent.

## Author contributions

MB was the principal investigator. DK was co-investigator. Both MB and DK collaborated together on all aspects of the design, implementation of IG exchange, and analysis.

### Conflict of interest statement

The authors declare that the research was conducted in the absence of any commercial or financial relationships that could be construed as a potential conflict of interest. The handling editor and reviewer ACC declared their involvement as co-editors in the Research Topic, and confirm the absence of any other collaboration.

## Footnotes

^1^It is interesting to note the choice of repertoire as representative pieces. The older adult choir focuses primarily on popular music with a special emphasis on current popular music. The children's choir was formed during the civil rights movement during the 1960's and will consistently add a traditional or popular song from this representative decade to teach from and perform in each concert.
